# Improvements during long-term fasting in patients with long COVID – a case series and literature review

**DOI:** 10.3389/fnut.2023.1195270

**Published:** 2023-11-02

**Authors:** Franziska Grundler, Robin Mesnage, Alberto Cerrada, Françoise Wilhelmi de Toledo

**Affiliations:** ^1^Buchinger Wilhelmi Clinic, Überlingen, Germany; ^2^Department of Medical and Molecular Genetics, King’s College London, London, United Kingdom; ^3^Buchinger Wilhelmi Clinic, Marbella, Spain

**Keywords:** long-term fasting, post-SARS-CoV-2 syndrome, long COVID, case report, well-being

## Abstract

**Background:**

Post-acute sequelae of a severe acute respiratory syndrome coronavirus 2 infection, also known as long COVID, comprises a variety of symptoms that impair the quality of life. This represents a growing public health burden, with millions of individuals worldwide affected.

**Case description:**

We present a case series of 14 COVID-19 patients with post-acute symptoms who underwent medically supervised long-term fasting (6 to 16 days) according to the peer-reviewed Buchinger Wilhelmi protocol. The EQ-5D-5L questionnaire and visual scales were used to evaluate the intensity of the symptoms, retrospectively during the acute phase, and prospectively before and after long-term fasting. Blood tests were also performed before and after fasting. Thirteen patients reported that fasting caused an enhancement in their perceived overall health. Only one patient had no improvement. Both frequent (fatigue, breathlessness, muscle and joint pains) and less frequent (cognitive impairment, smell and taste disorders) sequelae ameliorated. Body weight and other risk factors for cardiometabolic diseases like blood pressure, blood glucose, total cholesterol, low-density-lipoprotein cholesterol, and triglycerides were reduced. No severe side effects occurred.

**Discussion:**

This case series reports beneficial changes in self-perceived symptoms in patients with long COVID after long-term fasting. This highlights the potential of long-term fasting as an effective intervention for managing and treating long COVID.

## 1. Introduction

Long COVID, also known as “post-acute sequelae of COVID-19 (PASC),” is a condition that manifest after an incomplete recovery from a severe acute respiratory syndrome coronavirus 2 (SARS-CoV-2) infection in at least 10% of individuals (1). Symptoms are highly variable and can affect multiple systems, including respiratory, cardiovascular, neurological, gastrointestinal and musculoskeletal systems as well as mood disorders (2). The most frequent symptoms are fatigue, breathlessness, cognitive impairment and pain such as chest pain or headache (3, 4). Long COVID can develop in two phases depending on the persistence of symptoms. After the acute disease, ongoing symptoms can remain during 4 weeks to 3 months. If the symptoms persist for more than 3 months, the condition is classified as long COVID. According to the World Health Organisation (5), the symptoms of long COVID last for at least 2 months and cannot be explained by an alternative diagnosis. Thereby, the symptoms can either persist with possible fluctuations or emerge despite an initial recovery from an acute COVID-19 episode (5).

The SARS-CoV-2 virus primarily enters the cells through the angiotensin-converting enzyme 2 receptor, that is present in various cell types of the body, leading to an inflammatory response that impairs organ function (2). The underlying mechanisms of tissue damage in COVID-19 involve chronic inflammation (6, 7) and oxidative stress (8, 9), although we do not fully understand the mechanisms by which the infection leads to tissue damage. The subsequent development of long COVID is not fully predictable. Persistent viral antigens, the reactivation of human herpesviruses (e.g., Epstein–Barr), a higher virus load and the activation of autoreactive immunity are associated with immunologic perturbations that can predict the development of long COVID (1). Oxidative stress can lead to mitochondrial dysfunction, which impairs cellular energy homeostasis (8). The burst of reactive oxygen species caused by COVID-19 can lead to endothelial dysfunction. When this is combined with an elevated tendency for clotting, microclots can persist in plasma and block microcapillaries and thus impairing oxygen exchange (9). COVID-19-induced inflammation in the brain can result in dysfunctional neurological signaling and persist in neurological complications (1), including myelin loss (7) and tau hyperphosphorylation like in Alzheimer’s disease (6). Dysbiosis of the gut microbiota can even contribute to the pathogenesis of long COVID (10).

There is no consensus on therapeutic approaches to address long COVID (4). The multiplicity and complexity of symptoms of long COVID is incompatible with common pharmacological interventions, which are based on a one-symptom one-medication model. Non-pharmacological interventions like fasting or nutritional strategies act on multiple metabolic processes, ultimately leading to global changes in metabolic health (11).

Long-term fasting (LF) is defined as the voluntary renouncement of food intake lasting from 5 days to several weeks, depending on the individual physical condition (12). The Buchinger Wilhelmi fasting programme is minimally supplemented with up to 250 kcal through fruit juice and honey (13). This therapeutic approach has shown multiple effects on pathological mechanisms involved in COVID-19 infections, such as decreasing inflammation (14, 15) or reducing oxidative stress (16, 17). Furthermore, LF has been shown to reduce risk factors associated with severe COVID-19 courses. This includes weight loss, reduction in waist circumference (13), normalization of blood pressure (18), as well as glucose and lipid levels (13, 19) which are linked to COVID-19 comorbidities like obesity, hypertension, diabetes or dyslipidemia (2). Moreover, LF has been shown to affect gut microbiota (20) and improved well-being (13).

Up to now, clinical trials investigating the effects of LF on patients with long COVID are lacking. This case series provides the first documented improvements of self-reported symptoms and blood parameters before and after LF in patients with long COVID.

## 2. Methods

Regular patients of the Buchinger Wilhelmi clinics in Marbella (Spain) and Überlingen (Germany) with PSCA and confirmed positive PCR or SARS-CoV-2 rapid antigen test were included. All patients gave their written informed consent. Medical doctors conducted comprehensive medical history assessments, physical examinations before the fasting and documented height and waist circumference. Trained nurses measured every morning resting blood pressure, pulse, and body weight. Blood samples were collected before and after fasting. Adverse events were monitored continuously. For detailed descriptions, refer to (13), and Supplementary Figure S1 provides a visualization of the data collection.

The patients reported retrospectively about the acute phase of the COVID-19 infection. The severity level of the symptoms [asymptomatic, mild, moderate, severe, critical (life threatening)] and of the disease and level of care were determined.

Patients self-reported 19 symptoms that are potentially associated with COVID-19 on a visual analogue scale between 0 (none) and 10 (maximum) either retrospectively for the acute phase or prospectively before and after LF.

The EQ-5D-5L questionnaire was also applied for the acute phase, before and after LF. The questionnaire consists of five dimensions: mobility, self-care, usual activities, pain and anxiety/depression. Each dimension has five response levels: no problems, slight problems, moderate problems, severe problems, unable to /extreme problems. The index is calculated by applying a scoring algorithm (21). A value of 1 reflects full health, a value of 0 reflects worst health. The EQ-5D visual scale with the two endpoints “the best (100) and worst (0) health you can imagine” provides a quantitative measure of the perceived overall health (22).

Moreover, our intention was to ascertain which therapies or activities patients subjectively attributed the most to the improvement in their self-reported symptoms. Patients were provided with the option to denote their preferences by marking the categories of “fasting,” “diet,” “physiotherapy,” and “psychotherapy” or by providing individual comments under the “others” category. Two routine blood analysis were performed, one at the beginning and one at the end of fasting (13).

LF was performed according to the Buchinger Wilhelmi fasting program as described in details by Wilhelmi de Toledo et al. (13) and in line with the guidelines of fasting therapy (23).

## 3. Case description

Fourteen patients (5 women and 9 men) with persistent post-COVID-19 symptoms were admitted. Table 1 shows patients’ baseline characteristics and acute infection details. Age ranged from 33 (No. 9) to 74 years (No. 14). One patient (No. 11) was asymptomatic during the acute phase, while three had mild symptoms, five had moderate, and five had severe symptoms. The perception of acute symptoms varied widely from 1 day to 1.5 years. Five patients were able to perform their usual activities, three were unable to do so, and four were hospitalized during the acute phase. Three of them (Nos. 2, 12, 14) receiving oxygen treatment. The length of the inpatient stays was 3, 4, 5 and 10 days. Drugs were taken by five patients [corticosteroids (*n* = 4); antivirals (*n* = 3); antibiotics (*n* = 3)]. The period between acute infection and start of the fasting intervention varied from 5 to 22 months.

**Table 1:** Baseline characteristics of the cases and anamnesis before the fasting intervention.

No.	Sex	Age	COVID test	Symptom severity during acute infection	Duration of acute symptoms (days)	Disease severity and level of care	Hospitalization (days)	Drug treatment during acute phase	Time passed since the acute phase (weeks)	Clinic stay (days)	Fasting (days)	Food reintroduction (days)
1	M	68	Rapid	Mild	1				23	10	6	4
2	M	50	PCR	Severe		Hospital, supplemental oxygen	10	Corticosteroids; antibiotics	76	10	6	4
3	F	51	Rapid	Mild	5	No hospital, normal activities			26	15	12	3
4	M	69	Rapid	Moderate		No hospital, normal activities			96	14	6	8
5	M	51	PCR	Moderate	7	No hospital, normal activities			35	16	11	5
6	M	66	Rapid	Mild	3	No hospital, normal activities			53	12	8	4
7	F	58	PCR	Severe	144	Hospital, no oxygen	3		21	21	12	9
8	F	51	PCR	Severe	568	Hospital, no oxygen	0	Corticosteroids	81	11	10	1
9	F	33	PCR	Moderate	13	No hospital, no normal activities			31	13	9	4
10	M	60	Rapid	severe	9	No hospital, no normal activities			23	14	10	4
11	M	56	Rapid	Asymptomatic		No hospital, normal activities			39	21	16	5
12	M	45	Rapid	Severe	21	Hospital, supplemental oxygen	5	Antibiotics; antivirals	96	10	7	3
13	F	36	Rapid	Moderate		No hospital, no normal activities		Corticosteroids; antibiotics	68	11	8	3
14	M	74	Rapid	Moderate	9	Hospital, supplemental oxygen	4	Corticosteroids; antivirals	96	19	14	5

The patients had several pre-existing conditions that were diagnosed at the time of the fasting intervention. The most common diagnoses were obesity (*n* = 7), dyslipidemia (*n* = 4), digestive disorders (*n* = 4), and heart disease (*n* = 4). A table with all diagnoses per patient can be found in the supplements.

The patients stayed at the clinic for 10–21 days and underwent a fasting therapy for more than 6 days up to 16 days with a subsequent food reintroduction period of 3–5 days most patients (Table 1).

LF induced marked weight loss in all patients, as shown in Table 2. Systolic blood pressure decreased or remained unchanged, with patient No. 12, who had the highest initial value, experiencing the greatest reduction by −55 mmHg. Patient No. 9, who had the lowest initial value, showed no relevant change (−2 mmHg). Diastolic blood pressure changes followed a similar pattern, albeit less pronounced.

**Table 2:** Changes in body weight, BMI and blood pressure as well as glucose, lipid and inflammatory parameters before and after long-term fasting as well as calculated changes.

No.	Sex	Age (years)	BMI (kg/m^2^)	Fasting (days)	Height (cm)	Weight (kg)			BMI (kg/m^2^)			Systolic blood pressure (mmHg)			Diastolic blood pressure (mmHg)			Glucose (mg/dL)			Glycated hemoglobin (%)			Total cholesterol (mg/dL)			High-density cholesterol (mg/dL)			Low-density cholesterol (mg/dL)			Triglycerides (mg/dL)			Erythrocyte sedimentation rate (mm)			High-sensitivity reactive protein (mg/L)		
						Before	After	Change	Before	After	Change	Before	After	Change	Before	After	Change	Before	After	Change	Before	After	Change	Before	After	Change	Before	After	Change	Before	After	Change	Before	After	Change	Before	After	Change	Before	After	Change
1	M	68	35.3	6	181	115.5	109.2	−6.3	35.3	33.3	−1.9	140	130	−10	80	80	0	98	97	−1	5.5	5.3	−0.2	176	192	16	38	35	−3	112	135	23	131	112	−19	2	5	3	1.8	7.2	5.4
2	M	50	27.2	6	178	86.2	81.7	−4.5	27.2	25.8	−1.4	130	130	0	85	85	0	94	72	−22	6	6	0	209	196	−13	60	46	−14	130	129	−1	96	106	10	41	28	−13	46	7.3	−38.7
3	F	51	25.3	12	168	71.3	64.8	−6.5	25.3	23.0	−2.3	124	110	−14	80	70	−10	89	62	−27	5.3	5.1	−0.2	276	228	−48	71	54	−17	169	148	−21	181	131	−50	7	6	−1	1.2	2.6	1.4
4	M	69	34.4	6	182	113.9	110.5	−3.4	34.4	33.4	−1.0	120	104	−16	70	68	−2	90			5.3			183			59			108			80			4			<1		
5	M	51	33.1	11	191	120.8	112.1	−8.7	33.1	30.7	−2.4	120	110	−10	83	80	−3	121	82	−39	5.9	5.6	−0.3	205	144	−61	48	36	−12	139	98	−41	92	52	−40	10	13	3	<1	1.1	
6	M	66	32.0	8	181	104.9	99.4	−5.5	32.0	30.3	−1.7	146	137	−9	85	79	−6	104			5.4			226			58			141			136			3			1.1		
7	F	58	21.8	12	174	65.9	63.5	−2.4	21.8	21.0	−0.8	136	133	−3	83	81	−2	95	95	0	5.7	5.7	0	288	155	−133	47	38	−9	115	49	−66	134	136	2	2	2	0	0.5	0.2	−0.3
8	F	51	26.3	10	170	76.1	72.1	−4	26.3	25.0	−1.3	138	124	−14	86	84	−2	86	61	−25	5.2	5.1	−0.1	180	163	−17	75	59	−16	99	91	−8	55	79	24	2	2	0	0.9	3.9	3
9	F	33	21.7	9	176	67.1	60.8	−6.3	21.7	19.6	−2.1	100	98	−2	70	68	−2	131			5.2			164			54			95			155			2			<0.2		
10	M	60	28.6	10	180	92.7	85.0	−7.7	28.6	26.2	−2.4	131	118	−13	88	79	−9	104			6			176			37			131			100			2			1.5		
11	M	56	31.6	16	189	113.0	98.0	−15	31.6	27.4	−4.2	123	92	−31	74	64	−10	71	70	−1	4.7	4.4	−0.3	179	149	−30	48	63	15	111	70	−41	100	80	−20	3	4	1	<1	3.36	3.36
12	M	45	35.3	7	172	104.4	99.5	−4.9	35.3	33.6	−1.7	172	117	−55	89	75	−14	123	78	−45	6.3	6.1	−0.2	235	189	−46	47	47	0	163	131	−32	123	54	−69	6	5	−1	4.1	5.6	1.5
13	F	36	–	8	–	63.5	60.6	−2.9	–	–	–	100	99	−1	62	72	10	86	62	−24	5.1	5	−0.1	165	143	−22	77	59	−18	75	73	−2	63	55	−8	2	2	0	<1	<1	
14	M	74	32.0	14	170	92.6	82.9	−9.7	32.0	28.7	−3.4	132	120	−12	80	74	−6	85			5.4			236			43			153			199			9			1.1		

Blood test results before and after LF revealed a reduction in blood glucose levels (Table 2). However, glycated hemoglobin remained unchanged (Table 2). Lipid parameters were predominantly reduced (Table 2). Patients with high initial total cholesterol values had the strongest reduction (No. 7: −133 mg/dL; No. 5: −61 mg/dL). Inflammatory conditions were assessed using erythrocyte sedimentation rate (ESR), reflecting an indirect measurement of the inflammatory level in the body and high-sensitive C-reactive protein (hs CRP), reflecting a direct measurement of the inflammatory response. Changes in ESR were inconsistent. Three patients (No. 2, 3, 12), who had higher baseline levels, showed a reduction. Three patients (No. 1, 5, 11) had increased ESR levels after fasting and three patients with initial low values had no change. Hs-CRP level was reduced by −38,7 mg/L in patient No. 2 who had high initial values and in a second patient (No. 7). Five patients had increased hs-CRP levels after fasting. Additional results on blood count, blood markers, kidney and liver parameters as well as electrolytes are available in the Supplementary material.

**Figure 1:**
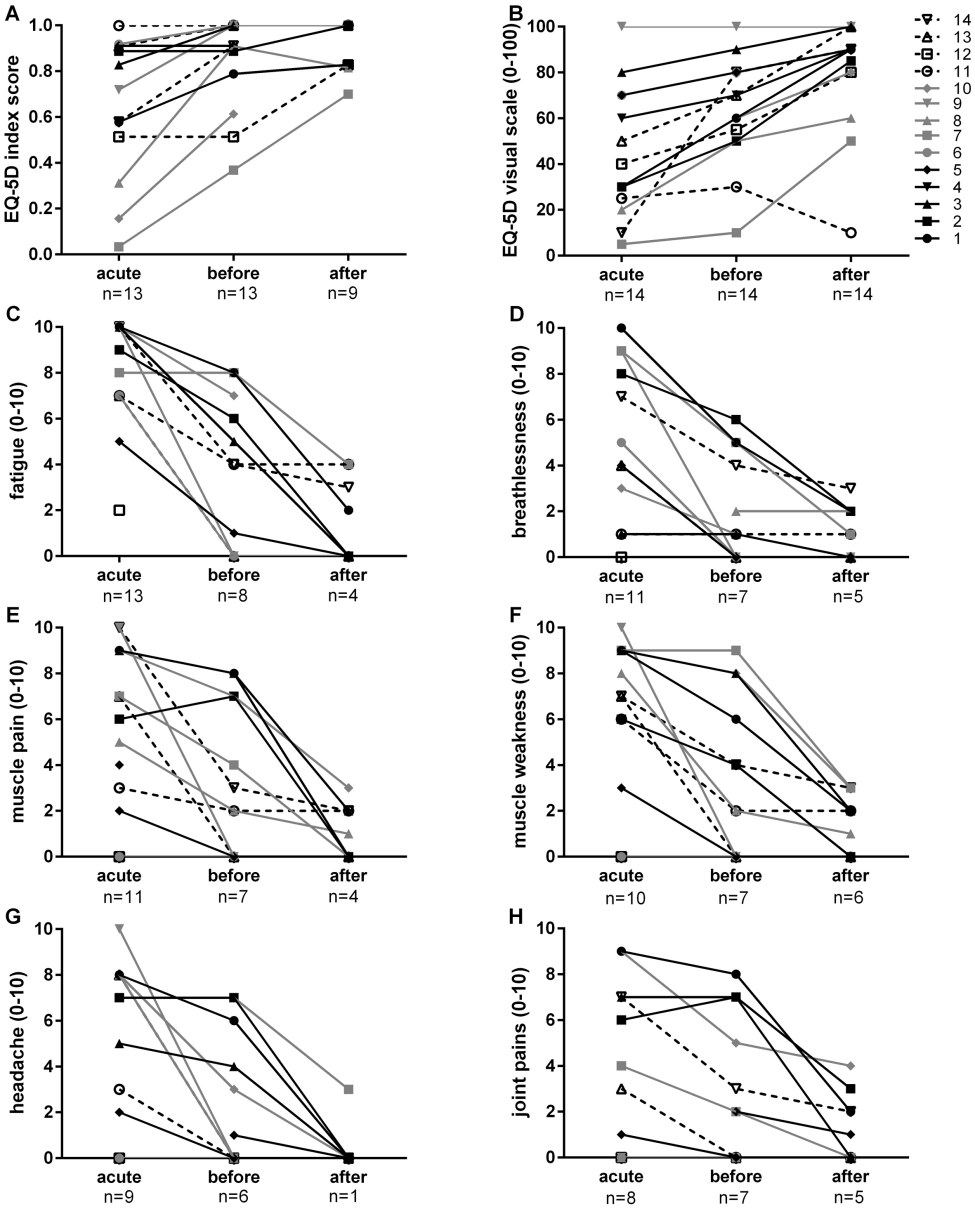
Changes in EQ-5D-5L index score **(A)** and visual scale **(B)** as well as most frequently mentioned self-reported symptoms (fatigue **C**; breathlessness **D**; muscle pain **E**; muscle weakness **F**; headache **G**; joint pains **H**) on a visual scale from 0 (none) to 10 (maximum). The EQ-5D-5L index score is based on the five dimensions mobility, self-care, usual activities, pain, and anxiety/depression. Each dimension has five response levels: no problems, slight problems, moderate problems, severe problems, unable to/extreme problems. The index is calculated by applying a scoring algorithm (21). A value of 1 reflects full health, a value of 0 reflects worst health. The EQ-5D visual scale provides a quantitative measure of the perceived overall health. The number of patients who answered (response > 0) is displayed below the time point.

Participants’ health perception based on the five dimensions mobility, self-care, usual activities, pain and anxiety/depression increased in most cases after the acute infection. Generally, the fasting intervention improved the health status. This is shown by the results of the EQ-5D index score and supported by the patients reported overall health on a visual analogue scale, that endpoints are labelled by “the best (100) and worst (0) health you can imagine” (Figure 1). In total, 13 patients indicated an improvement.

**Figure 2:**
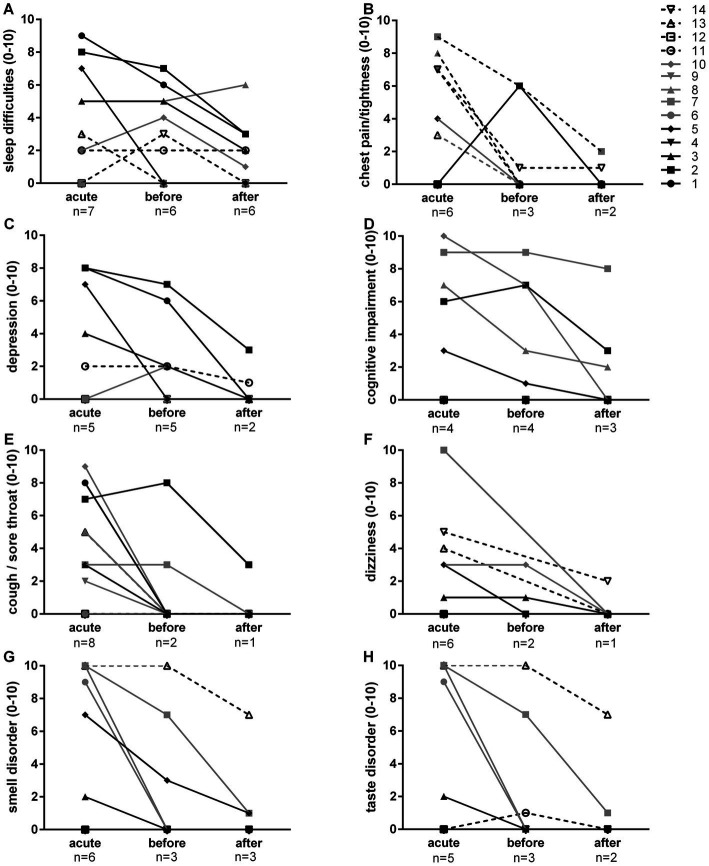
Self-reported symptoms (sleep difficulties, **A**; chest pain/tightness, **B**; depression, **C**; cognitive impairment, **D**; cough/sore throat, **E**; dizziness, **F**; smell disorder, **G**; taste disorder, **H**) on a visual scale from 0 (none) to 10 (maximum). The number of patients that reported to experience the symptom (response > 0) are indicated below for the acute phase, as well as before and after long-term fasting.

Figures 1C–H, 2A–H show symptom intensity on a visual scale from 0 (none) to 10 (maximum). The most common symptoms were fatigue, breathlessness, muscle pain, muscle weakness, headache, joint pains, sleep difficulties and chest pain/tightness. Improvement was reported by many patients after the acute phase. Ongoing symptoms could be further improved by LF in the majority of the cases (Figure 1). Less frequent post-acute sequelae such as depression, cognitive impairment, cough/sore throat, dizziness as well as smell and taste disorders improved in all after LF (Figure 2). While fever was predominantly a symptom during the acute phase, abdominal pain, skin rash, diarrhea, and nausea were rare. Nevertheless, improvement was observed during LF (Supplementary Figure S2).

Patients were asked about their opinion, which therapies and activities helped the most to improve their symptoms. Out of the 14 patients, 10 mentioned fasting as the most helpful treatment. Six patients found physiotherapy helpful. Other treatments mentioned included ozone therapy, acupuncture, psychotherapy, and osteopathy. No adverse events were observed.

## 4. Discussion

We report the beneficial effects of LF in 14 patients suffering from sequelae of COVID-19. Perceived overall health was enhanced, with improvements in common symptoms like fatigue, breathlessness, muscle pain and weakness, headache, joint pains and sleep difficulties. Even less frequent, but classical COVID symptoms, like cognitive impairments, smell and taste disorders were ameliorated. Given the lack of therapeutic approaches to treat long COVID, our observations suggests that long-term fasting could be a non-pharmacological approach to treat long COVID. This is corroborated by a large number of studies showing that fasting targets multiple aspects of long COVID, which are elaborated upon in the following discussion.

Voluntarily renunciation of food intake during an acute infection is a known behavioral adaptation to enhance survival (24). Clinical experience and case reports have described the positive effects of fasting on infectious diseases such as typhoid and scarlet fever, or tonsillitis (25). Even controlled laboratory studies showed that when mice were infected with *Listeria monocytogenes*, 95% of normally fed mice died while only 5% of the previously starved mice died (26). This enhancement of survival can be linked to adaptive defense mechanisms that are triggered by fasting. When food intake is interrupted, the body switches to fat burning as primary energy source, leading to the production of ketone bodies. Recent research has demonstrated that nutritional ketosis can promote antiviral immunity in lung (27). The ketone body β-hydroxybutyrate directly increases CD8+ T effector cell cytokine production through effects on histone acetylation (28).

The known beneficial effects of fasting on brain metabolism could provide explanations for the enhancement in self-perceived health observed in this study. In the rodent brain, fasting reduced oxidative stress (29) and enhanced mitochondrial oxidative metabolism (30). This was linked to the activity of the ketone body beta-hydroxybutyrate (BHB) which improves brain function by triggering the releasing brain-derived neurotrophic factor, increasing cognitive performance, memory, synaptic plasticity, neurogenesis and resistance to injury and diseases (31). Fasting is even known to cause a pronounced stimulation of the hypothalamic–pituitary–adrenal axis conferring a pain-relieving effect (32). These fasting-induced hormonal changes could explain lower muscle and joint pain as well as headache found in this study. The marked improvements in muscle pain and weakness are supported by a study that showed an increased in leg muscle power and strength during a 10-days fast (15). In a larger group of 1,422 persons fasting for 4 to 21 days, fasting caused an elevation in emotional and physical well-being and increased energy levels (13, 18).

Furthermore, Fasting affects coagulation parameters in adults (13), potentially counteracting clotting tendency during PASC. Moreover, fasting promotes vasodilatation through adiponectin-mediated endothelial nitric oxide production in rats (33). This could mitigate endothelial dysfunction. Sleep quality increased after 7 fasting days which aligns with the observed reduction in sleep difficulties in other studies (34).

The cessation of food intake triggers the metabolic switch from glucose to fat and ketone utilization, which is reflected at the molecular level by the inactivation of the nutrient sensitive mammalian target of rapamycin (mTOR) pathway. It could be speculated that the effects of fasting on COVID are mediated by mTOR. Furthermore, mTOR signalling can activate the inflammasome, which might be associated with the cytokine storm during COVID The hyper-production of cytokines during COVID occurs especially in obese (35), where the mTOR signaling is already elevated and linked to weight gain and insulin resistance (36). This could be why obesity with its chronic inflammation is a risk factor for severe COVID-19 outcomes characterized by a strong inflammatory response in the adipose tissue (37, 38).

The dysregulation of the immune function is a hallmark of COVID-19. Fasting could possibly boost the antiviral immune function and survival of activated T cells via the production of BHB which has been showed to serve as a more efficient alternative fuel for T cells (27). This is supported by demonstrations that ketogenic diets have been reported to ameliorate clinical symptoms of pulmonary health conditions (39). A way to reduce inflammation is to diminish the volume of adipose tissue by a fasting-induced weight loss, as observed in the presented cases and previous studies, as well as reducing visceral fat (13). Further anti-inflammatory mechanisms could be triggered by activation of the Nuclear factor erythroid 2-related factor (Nrf)-2/heme oxygenase (HO)-1 signaling pathway (40). The anti-inflammatory effect of fasting is documented in inflammatory diseases like rheumatoid arthritis (14) and shown by the decrease of various parameters like tumor necrosis factor-α, or interleukin-6 (15). Additionally, the inactivation of the mTOR cascade promotes autophagy (41). This may help to clear viral RNA through lysosomes (42) and repair post-acute illness damages (43).

Fasting can improve cellular stress adaptation by eliminating free radicals. A 10-days fast increased total antioxidant capacity and diminished damages from lipid peroxidation (16, 17). Asthmatic patients had less oxidative stress after 8-weeks alternate day fasting (44). Moreover, cells switch during fasting into a protective mode, which prevents DNA damages and induces DNA repair (45). In mice, fasting activated hematopoietic stem cells, enhancing self-renewal and lineage-balanced regeneration of immune cells (46). These findings suggest that fasting could reverse immunosuppressive states and strengthen the innate immune system.

Patients suffering from COVID-19 have an increased risk to develop cardiovascular diseases (47). Furthermore, alternations in lipid profile of COVID-19 patients have been demonstrated (48). Fasting studies have shown both preventive and therapeutic improvements in glucose and insulin levels (15), cholesterol and triglyceride levels (19), high blood pressure (18), and fatty liver (49). The presented cases also displayed improvements in blood results and blood pressure measurements.

Studies have linked microbiome dysbiosis to SARS-CoV-2-infections and increased mortality (50). Fasting has been shown to influence gut microbiome composition, increased short chain fatty acid production, and reduce gut permeability (20). The influence on health effects during PSAC require further investigation.

The safety of fasting during acute virus infections raises concerns due to contradicting findings in mouse experiments. While fasting improved survival in models of bacterial inflammation, glucose utilization was found to be crucial for survival in models of viral inflammation (51). More studies are needed to assess the safety of fasting during an acute infection. Furthermore, it will be of importance to determine the optimal period for initiating fasting therapy after an acute infection as well as to determine the optimal fasting length. This case series is limited by the reliance on retrospective data of the acute phase, subjective evaluations, missing data, the lack of viral load assessment and consideration of other confounders. Future studies should also incorporate a non-fasting control group to enable the extrapolation of the effectiveness of the fasting treatment.

Altogether, an increasing number of studies showed that LF triggers well-orchestrated processes like autophagy, oxidative stress defense, anti-inflammatory responses, contributing to a normalization of the metabolism and improvements in cardiovascular risk factors. In long COVID patients, these effects could potentially restore organ function, decrease systemic inflammation and oxidative stress, and ultimately recover the health status. While further research is warranted, the improved well-being and reduced physical complaints observed in long COVID patients fasting at the Buchinger Wilhelmi Clinic suggests that LF could be a potent a non-pharmacological approach in reclaiming health after the contraction of serious infectious diseases.

## Data availability statement

The original contributions presented in the study are included in the article/Supplementary material, further inquiries can be directed to the corresponding author.

## Ethics statement

Written informed consent was obtained from the individual(s) for the publication of any potentially identifiable images or data included in this article. Written informed consent was obtained from the participant/patient(s) for the publication of this case report.

## Author contributions

FG: conceptualization, data collection, writing original draft, and preparation. RM: writing – review and editing. AC: data collection. FW: conceptualization, and writing – review and editing. All authors contributed to the article and approved the submitted version.

## Supplementary material

Supplementary Figure S1 Visualisation of the data collection.

Supplementary Figure S2 Self-reported symptoms, fever **(A)**, abdominal pain **(B)**, skin rash **(C)**, diarrhea **(D)** and nausea **(E)**, on a visual scale from 0 (none) to 10 (maximum). The number of patients that reported to experience the symptom (response > 0) are indicated below for the acute phase, as well as before and after long-term fasting.
