# Supplementary figures and images for: Improvements during long-term fasting in patients with long COVID – a case series and literature review
Source: Front Nutr. 2023 Nov 2;10:1195270. doi: 10.3389/fnut.2023.1195270 (PMC10651743; doi:10.3389/fnut.2023.1195270)

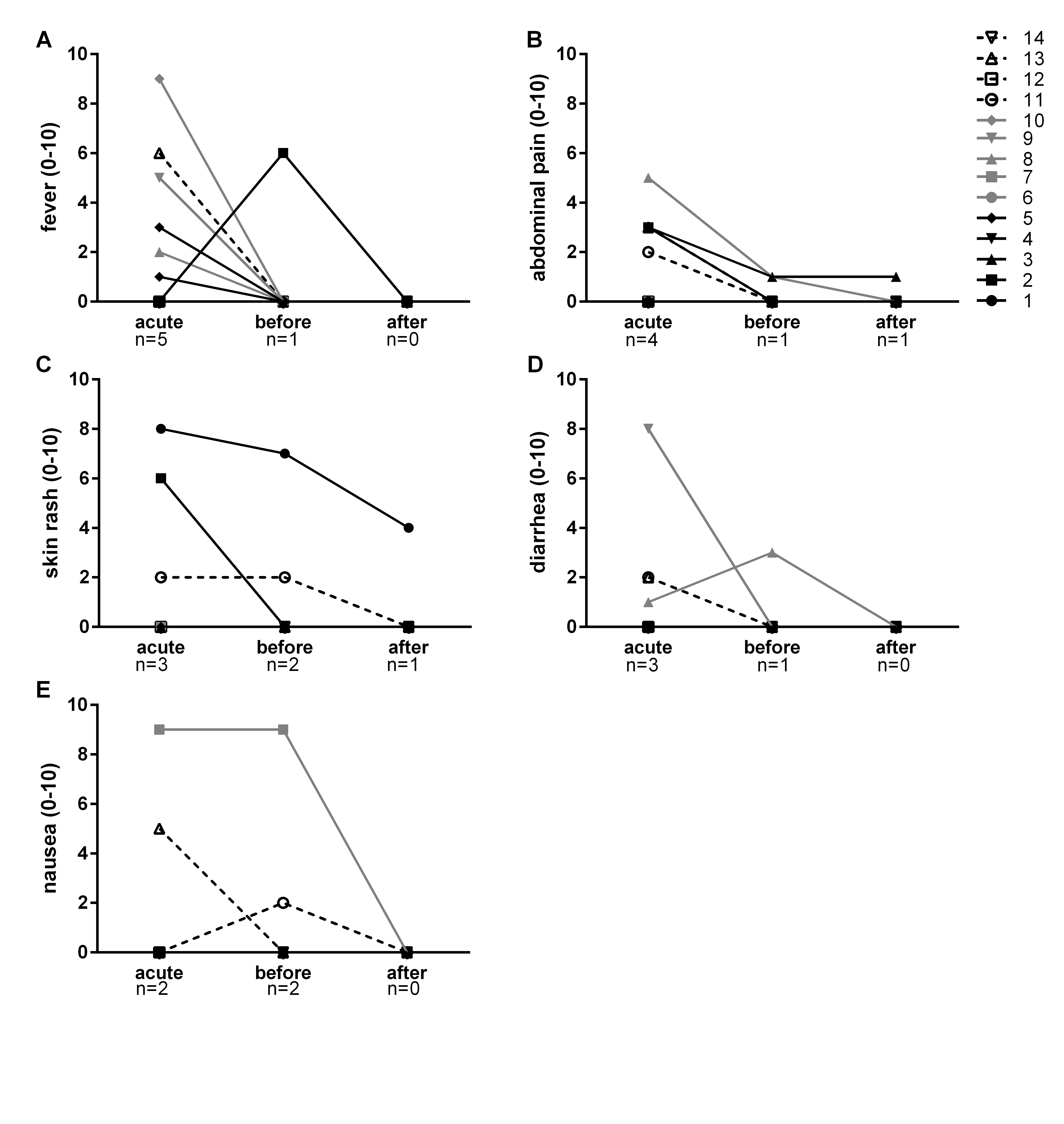

Supplement: Supplementary Figure S1 — Visualisation of the data collection. [file Image_1.JPEG]

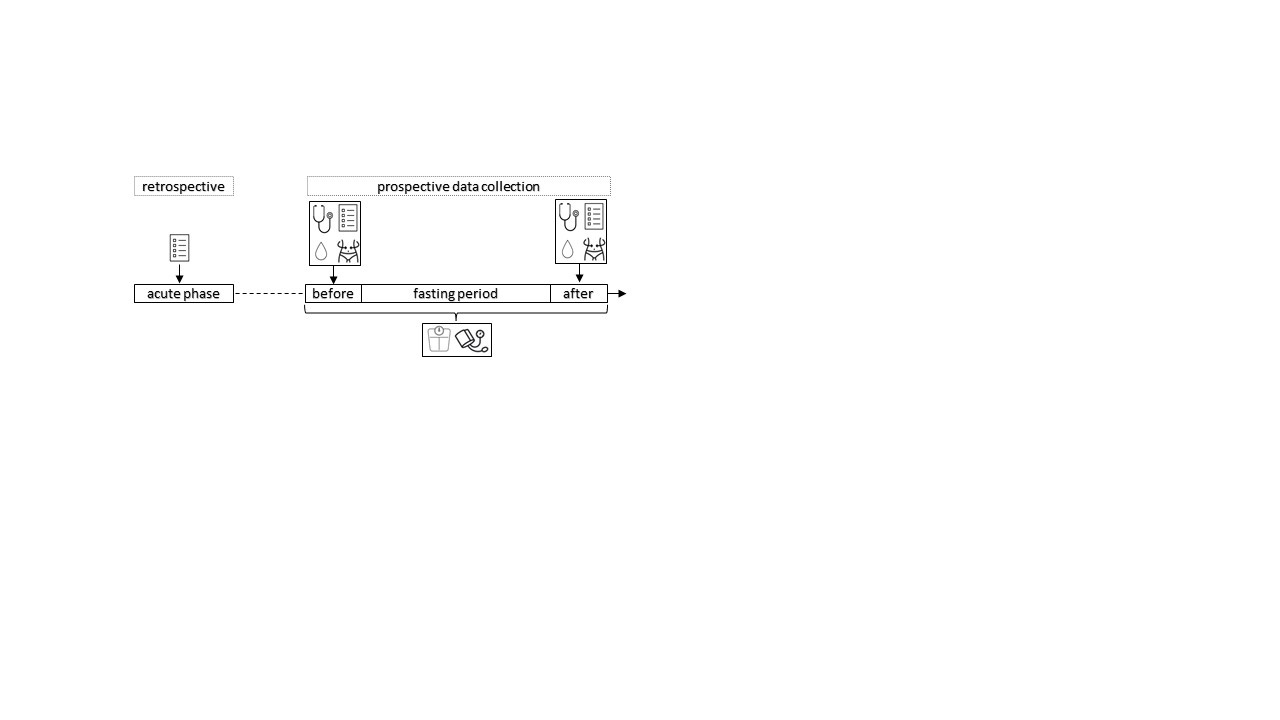

Supplement: Supplementary Figure S2 — Self-reported symptoms, fever (A), abdominal pain (B), skin rash (C), diarrhea (D) and nausea (E), on a visual scale from 0 (none) to 10 (maximum). The number of patients that reported to experience the symptom (response > 0) are indicated below for the acute phase, as well as before and after long-term fasting. [file Image_2.JPEG]
