# Supplementary material for: Improvements during long-term fasting in patients with long COVID – a case series and literature review
Source: Front Nutr. 2023 Nov 2;10:1195270. doi: 10.3389/fnut.2023.1195270 (PMC10651743; doi:10.3389/fnut.2023.1195270)
Supplement: Supplementary file 3 [file Data_Sheet_1.docx]

Supplementary Material

**Improvements during long-term fasting in patients with long COVID**

**– a case series and literature review**

Franziska Grundler^1^*, Robin Mesnage^1,2^, Alberto Cerrada^3^, Françoise Wilhelmi de Toledo^1^

*** Correspondence:** Dr. Franziska Grundler: franziska.grundler@buchinger-wilhelmi.com

# Supplementary Figures and Tables

**Figure S1. Visualisation of the data collection.**
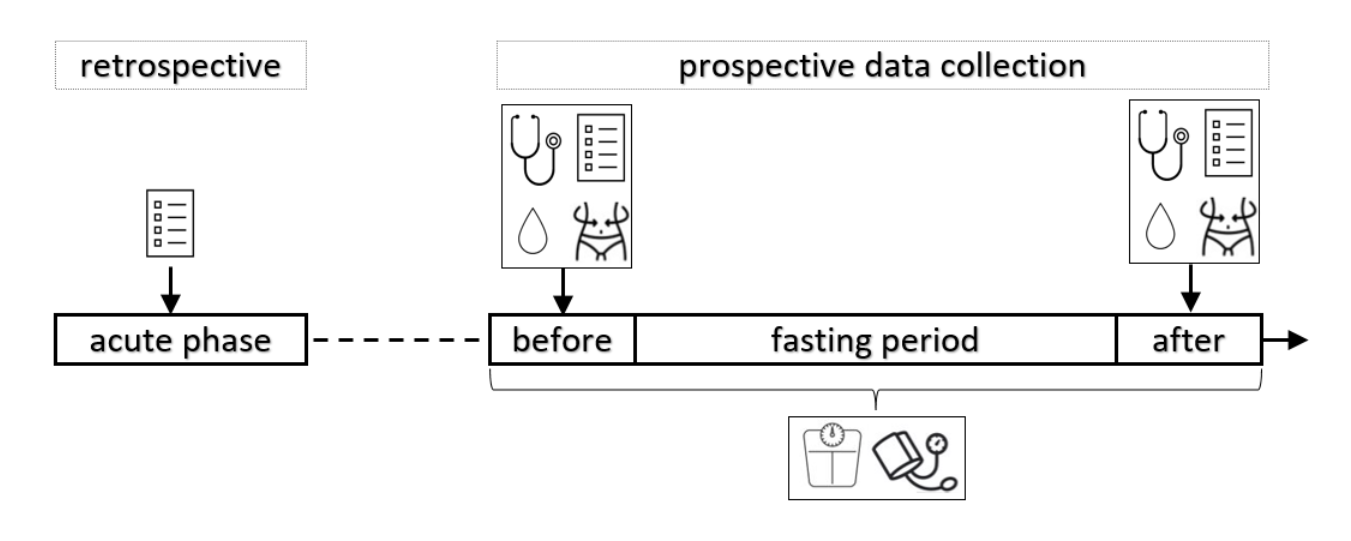


**Figure S2. Rarely mentioned self-reported symptoms (fever, A; abdominal pain, B; skin rash, C; diarrhea, D; nausea, E) on a visual scale from 0 (none) to 10 (maximum).** The number of patients that reported to experience the symptom (response > 0) are indicated below for the acute phase, as well as before and after long-term fasting.

**Table S1.** Diagnosis of the patients.

| **No.** | **sex** | **age (years)** | **fasting (days)** | **Obesity** | **Digestive probelms** | **Dyslipidemia** | **Allergies** | **Heart disease** | **Sleep disorders** | **Vitamin D deficiency** | **Autoimmune disease** | **Asthma** | **Hyperplasie** | **Hyperuricemia** | **Hiatal hernia** | **Menopause** | **Taste disorder** | **Migraine** | **Diabetes** | **Hepatic steatosis** | **Hypertension** | **Headache** | **Smell disorder** | **Others** |
| --- | --- | --- | --- | --- | --- | --- | --- | --- | --- | --- | --- | --- | --- | --- | --- | --- | --- | --- | --- | --- | --- | --- | --- | --- |
| **1** | M | 68 | 6 | x |  |  |  |  |  |  |  |  |  |  |  |  |  |  |  |  |  |  |  | Lumbar disc disorder |
| **2** | M | 50 | 6 |  |  | x |  |  |  | x |  |  |  |  |  |  |  |  |  |  |  |  |  |  |
| **3** | F | 51 | 12 |  | x | x |  |  |  | x |  |  |  |  |  |  |  |  |  |  |  |  |  |  |
| **4** | M | 69 | 6 | x | x | x |  | x |  |  |  |  |  |  | x |  |  |  |  |  |  | x |  | Idiopathic urticaria |
| **5** | M | 51 | 11 | x | x |  | x |  |  |  |  |  |  |  | x |  |  |  |  | x |  |  |  |  |
| **6** | M | 66 | 8 | x |  |  |  | x | x |  |  |  | x |  |  |  |  |  |  |  | x |  |  |  |
| **7** | F | 58 | 12 |  |  |  |  | x |  |  |  |  |  |  |  | x | x |  |  |  |  |  |  | Herpes simplex, memory disorder, loss of appetite, nausea |
| **8** | F | 51 | 10 |  | x |  |  |  | x |  | x | x |  |  |  | x |  |  |  |  |  |  |  | Psoriasis vulgaris |
| **9** | F | 33 | 9 |  |  |  |  |  | x |  |  |  |  |  |  |  |  | x |  |  |  |  |  |  |
| **10** | M | 60 | 10 |  |  |  | x |  |  |  | x | x |  |  |  |  |  |  |  |  |  |  |  |  |
| **11** | M | 56 | 16 | x |  |  |  |  |  |  |  |  |  |  |  |  |  |  |  |  |  |  |  |  |
| **12** | M | 45 | 7 | x |  | x |  |  |  | x |  |  |  | x |  |  |  |  | x |  |  |  |  |  |
| **13** | F | 36 | 8 |  |  |  |  |  |  |  |  |  |  |  |  |  | x |  |  |  |  |  | x |  |
| **14** | M | 74 | 14 | x |  |  |  | x |  |  |  |  | x | x |  |  |  |  |  |  |  |  |  | Internal knee disorder, cataract, aspiration |

**Table S2.** Blood count and blood cell markers before and after long-term fasting as well as calculated changes.

| **No.** | **sex** | **age (years)** | **BMI (kg/m^2)^** | **fasting (days)** | **Erythrocytes** | **(x10^6^/mm³)** |  | **Mean corpuscular volume** | **(fL)** |  | **Mean corpuscular hemoglobin** | **(pg)** |  | **Mean corpuscular hemoglobin concentration** | **(g/dL)** |  | **Hemoglobin** | **(g/dL)** |  | **Hematocrite** | **(%)** |  | **Leucocytes** | **(x10³/mm³)** |  | **Thrombozytes** | **(x10³/mm³)** |  |
| --- | --- | --- | --- | --- | --- | --- | --- | --- | --- | --- | --- | --- | --- | --- | --- | --- | --- | --- | --- | --- | --- | --- | --- | --- | --- | --- | --- | --- |
|  |  |  |  |  | **before** | **after** | **change** | **before** | **after** | **change** | **before** | **after** | **change** | **before** | **after** | **change** | **before** | **after** | **change** | **before** | **after** | **change** | **before** | **after** | **change** | **before** | **after** | **change** |
| **1** | M | 68 | 35.3 | 6 | 3.65 | 4.89 | 1.24 | 95 | 94 | -1 | 36.4 | 31.9 | -4.5 | 38.4 | 34.1 | -4.3 | 13.3 | 15.6 | 2.3 | 34.6 | 45.8 | 11.2 | 5.2 | 6.4 | 1.2 | 129 | 196 | 67 |
| **2** | M | 50 | 27.2 | 6 | 4.65 | 4.87 | 0.22 | 91 | 90 | -1 | 28.6 | 28.7 | 0.1 | 31.6 | 32.1 | 0.5 | 13.3 | 14 | 0.7 | 42.1 | 43.6 | 1.5 | 9.3 | 8.2 | -1.1 | 284 | 322 | 38 |
| **3** | F | 51 | 25.3 | 12 | 4.63 | 4.79 | 0.16 | 88 | 86 | -2 | 27.2 | 28 | 0.8 | 30.9 | 32.6 | 1.7 | 12.6 | 13.4 | 0.8 | 40.8 | 41.1 | 0.3 | 8.3 | 5.6 | -2.7 | 382 | 392 | 10 |
| **4** | M | 69 | 34.4 | 6 | 4.51 |  |  | 90 |  |  | 28.6 |  |  | 31.9 |  |  | 12.9 |  |  | 40.4 |  |  | 4.9 |  |  | 250 |  |  |
| **5** | M | 51 | 33.1 | 11 | 4.79 | 4.65 | -0.14 | 96 | 96 | 0 | 32.2 | 32.3 | 0.1 | 33.4 | 33.6 | 0.2 | 15.4 | 15 | -0.4 | 46.1 | 44.7 | -1.4 | 7.2 | 4.3 | -2.9 | 234 | 216 | -18 |
| **6** | M | 66 | 32.0 | 8 | 4.74 |  |  | 97 |  |  | 33.1 |  |  | 34.1 |  |  | 15.7 |  |  | 46.1 |  |  | 6.5 |  |  | 190 |  |  |
| **7** | F | 58 | 21.8 | 12 | 4.61 | 4.25 | -0.36 | 88.3 | 87.3 | -1 | 29.9 | 30.4 | 0.5 | 33.9 | 34.8 | 0.9 | 13.8 | 12.9 | -0.9 | 40.7 | 37.1 | -3.6 | 6.6 | 4.8 | -1.8 | 228 | 198 | -30 |
| **8** | F | 51 | 26.3 | 10 | 4.86 | 4.71 | -0.15 | 89.9 | 88.3 | -1.6 | 31.3 | 31.2 | -0.1 | 34.8 | 35.3 | 0.5 | 15.2 | 14.7 | -0.5 | 43.7 | 41.6 | -2.1 | 5.6 | 5.8 | 0.2 | 253 | 249 | -4 |
| **9** | F | 33 | 21.7 | 9 | 3.78 |  |  | 95.2 |  |  | 32.8 |  |  | 34.4 |  |  | 12.4 |  |  | 36 |  |  | 7.1 |  |  | 267 |  |  |
| **10** | M | 60 | 28.6 | 10 | 4.69 |  |  | 91.5 |  |  | 32.4 |  |  | 35.4 |  |  | 15.2 |  |  | 42.9 |  |  | 6 |  |  | 222 |  |  |
| **11** | M | 56 | 31.6 | 16 | 4.58 | 4.89 | 0.31 | 90 | 88 | -2 | 30.3 | 31.1 | 0.8 | 33.7 | 35.3 | 1.6 | 13.9 | 15.2 | 1.3 | 41.3 | 43.1 | 1.8 | 5.7 | 5.6 | -0.1 | 229 | 256 | 27 |
| **12** | M | 45 | 35.3 | 7 | 5.37 | 5.58 | 0.21 | 89 | 87 | -2 | 29.6 | 29.4 | -0.2 | 33.4 | 33.8 | 0.4 | 15.9 | 16.4 | 0.5 | 47.6 | 48.5 | 0.9 | 7.8 | 7 | -0.8 | 235 | 240 | 5 |
| **13** | F | 36 | ̶ | 8 | 4.25 | 4.16 | -0.09 | 97 | 96 | -1 | 32 | 31.7 | -0.3 | 32.9 | 33 | 0.1 | 13.6 | 13.2 | -0.4 | 41.3 | 40 | -1.3 | 4.9 | 3.8 | -1.1 | 223 | 202 | -21 |
| **14** | M | 74 | 32.0 | 14 | 4.5 |  |  | 95 |  |  | 31.8 |  |  | 33.3 |  |  | 14.3 |  |  | 42.9 |  |  | 5.4 |  |  | 190 |  |  |

**Table S3.** Differential blood count before and after long-term fasting as well as calculated changes.

| **No.** | **Sex** | **age (years)** | **BMI (kg/m^2)^** | **fasting (days)** | **Basophiles** | **(%)** |  | **Basophiles** | **(x10³/mm³)** |  | **Eosinophiles** | **(%)** |  | **Eosinophiles** | **(x10³/mm³)** |  | **Lymphozytes** | **(x10³/mm³)** |  | **Lymphocytes** | **(%)** |  |
| --- | --- | --- | --- | --- | --- | --- | --- | --- | --- | --- | --- | --- | --- | --- | --- | --- | --- | --- | --- | --- | --- | --- |
|  |  |  |  |  | **before** | **after** | **change** | **before** | **after** | **change** | **before** | **after** | **change** | **before** | **after** | **change** | **before** | **after** | **change** | **before** | **after** | **change** |
| **1** | M | 68 | 35.3 | 6 | 0.6 | 0.8 | 0.2 | 0 | 0.1 | 0.1 | 4.2 | 2.8 | -1.4 | 0.2 | 0.2 | 0 | 1.7 | 2.2 | 0.5 | 32.6 | 34.7 | 2.1 |
| **2** | M | 50 | 27.2 | 6 | 0.9 | 0.9 | 0 | 0.1 | 0.1 | 0 | 19.5 | 12.2 | -7.3 | 1.8 | 1 | -0.8 | 3.3 | 2.5 | -0.8 | 35.9 | 30.8 | -5.1 |
| **3** | F | 51 | 25.3 | 12 | 0.6 | 1.1 | 0.5 | 0.1 | 0.1 | 0 | 2.3 | 2.5 | 0.2 | 0.2 | 0.1 | -0.1 | 3.7 | 2.4 | -1.3 | 43.9 | 42.5 | -1.4 |
| **4** | M | 69 | 34.4 | 6 | 0.8 |  |  | 0 |  |  | 4.5 |  |  | 0.2 |  |  | 2 |  |  | 41.3 |  |  |
| **5** | M | 51 | 33.1 | 11 | 0.7 | 0.7 | 0 | 0.1 | 0 | -0.1 | 5.7 | 4.4 | -1.3 | 0.4 | 0.2 | -0.2 | 2.4 | 1.4 | -1 | 32.8 | 32.6 | -0.2 |
| **6** | M | 66 | 32.0 | 8 | 0.3 |  |  | 0 |  |  | 4 |  |  | 0.3 |  |  | 2.6 |  |  | 39.6 |  |  |
| **7** | F | 58 | 21.8 | 12 |  |  |  |  |  |  |  |  |  |  |  |  |  |  |  |  |  |  |
| **8** | F | 51 | 26.3 | 10 |  |  |  |  |  |  |  |  |  |  |  |  |  |  |  |  |  |  |
| **9** | F | 33 | 21.7 | 9 |  |  |  |  |  |  |  |  |  |  |  |  |  |  |  |  |  |  |
| **10** | M | 60 | 28.6 | 10 |  |  |  |  |  |  |  |  |  |  |  |  |  |  |  |  |  |  |
| **11** | M | 56 | 31.6 | 16 | 0.7 | 0.5 | -0.2 | 0 | 0 | 0 | 1.9 | 1.1 | -0.8 | 0.1 | 0.1 | 0 | 1.9 | 1.8 | -0.1 | 33.5 | 32 | -1.5 |
| **12** | M | 45 | 35.3 | 7 | 0.5 | 0.6 | 0.1 | 0 | 0 | 0 | 2.3 | 2.4 | 0.1 | 0.2 | 0.2 | 0 | 2.5 | 1.7 | -0.8 | 32.5 | 23.6 | -8.9 |
| **13** | F | 36 | ̶ | 8 | 0.6 | 0.8 | 0.2 | 0 | 0 | 0 | 3.3 | 1.1 | -2.2 | 0.2 | 0 | -0.2 | 2.1 | 1.8 | -0.3 | 42.2 | 46.8 | 4.6 |
| **14** | M | 74 | 32.0 | 14 | 0.7 |  |  | 0 |  |  | 2.4 |  |  | 0.1 |  |  | 2.3 |  |  | 43.6 |  |  |

**Table S4.** Differential blood count and cell markers before and after long-term fasting as well as calculated changes.

| **No.** | **Sex** | **age (years)** | **BMI (kg/m^2)^** | **fasting (days)** | **Monocytes** | **(%)** |  | **Monocytes** | **(x10³/mm³)** |  | **Neutrophiles** | **(%)** |  | **Neutrophiles** | **(x10³/mm³)** |  | **Red cell distribution width** | **(%)** |  | **Mean platelet volume** | **(fL)** |  |
| --- | --- | --- | --- | --- | --- | --- | --- | --- | --- | --- | --- | --- | --- | --- | --- | --- | --- | --- | --- | --- | --- | --- |
|  |  |  |  |  | **before** | **after** | **change** | **before** | **after** | **change** | **before** | **after** | **change** | **before** | **after** | **change** | **before** | **after** | **change** | **before** | **after** | **change** |
| **1** | M | 68 | 35.3 | 6 | 7.7 | 9.2 | 1.5 | 0.4 | 0.6 | 0.2 | 54.9 | 52.5 | -2.4 | 2.9 | 3.4 | 0.5 | 13 | 12.8 | -0.2 | 10.1 | 10 | -0.1 |
| **2** | M | 50 | 27.2 | 6 | 9.2 | 10.9 | 1.7 | 0.9 | 0.9 | 0 | 34.5 | 45.2 | 10.7 | 3.2 | 3.7 | 0.5 | 14.5 | 14.3 | -0.2 | 11.7 | 11.8 | 0.1 |
| **3** | F | 51 | 25.3 | 12 | 7.5 | 10.4 | 2.9 | 0.6 | 0.6 | 0 | 45.7 | 43.5 | -2.2 | 3.8 | 2.4 | -1.4 | 18.8 | 19 | 0.2 | 10.7 | 10.7 | 0 |
| **4** | M | 69 | 34.4 | 6 | 9.3 |  |  | 0.5 |  |  | 44.1 |  |  | 2.2 |  |  | 13.1 |  |  | 10.1 |  |  |
| **5** | M | 51 | 33.1 | 11 | 9.3 | 10.3 | 1 | 0.7 | 0.4 | -0.3 | 51.5 | 52 | 0.5 | 3.7 | 2.2 | -1.5 | 12.6 | 12.1 | -0.5 | 11 | 11.4 | 0.4 |
| **6** | M | 66 | 32.0 | 8 | 9.3 |  |  | 0.6 |  |  | 46.8 |  |  | 3 |  |  | 12.2 |  |  | 9.7 |  |  |
| **7** | F | 58 | 21.8 | 12 |  |  |  |  |  |  |  |  |  |  |  |  | 12 | 13 | 1 |  |  |  |
| **8** | F | 51 | 26.3 | 10 |  |  |  |  |  |  |  |  |  |  |  |  | 12 | 12 | 0 |  |  |  |
| **9** | F | 33 | 21.7 | 9 |  |  |  |  |  |  |  |  |  |  |  |  | 14 |  |  |  |  |  |
| **10** | M | 60 | 28.6 | 10 |  |  |  |  |  |  |  |  |  |  |  |  | 13 |  |  |  |  |  |
| **11** | M | 56 | 31.6 | 16 | 9.2 | 11 | 1.8 | 0.5 | 0.6 | 0.1 | 54.7 | 55.4 | 0.7 | 3.1 | 3.1 | 0 | 13.1 | 13 | -0.1 | 10.4 | 10.5 | 0.1 |
| **12** | M | 45 | 35.3 | 7 | 6.9 | 7 | 0.1 | 0.5 | 0.5 | 0 | 57.8 | 66.4 | 8.6 | 4.5 | 4.6 | 0.1 | 13.1 | 12.1 | -1 | 12.7 | 12.6 | -0.1 |
| **13** | F | 36 | ̶ | 8 | 9.8 | 11.4 | 1.6 | 0.5 | 0.4 | -0.1 | 44.1 | 39.9 | -4.2 | 2.2 | 1.5 | -0.7 | 11.9 | 11.6 | -0.3 | 11.9 | 12.6 | 0.7 |
| **14** | M | 74 | 32.0 | 14 | 11 |  |  | 0.6 |  |  | 42.3 |  |  | 2.3 |  |  | 12.7 |  |  | 10.7 |  |  |

**Table S5.** Kidney and liver parameters before and after long-term fasting as well as calculated changes.

| **No.** | **Sex** | **age (years)** | **BMI (kg/m^2)^** | **fasting (days)** | **uric acid** | **(mg/dL)** |  | **urea** | **(mg/dL)** |  | **creatinine** | **(mg/dL)** |  | **aspartate aminotransferase** | **(U/L)** |  | **alanine aminotransferase** | **(U/L)** |  | **gamma-glutamyl transferase** | **(U/L)** |  |
| --- | --- | --- | --- | --- | --- | --- | --- | --- | --- | --- | --- | --- | --- | --- | --- | --- | --- | --- | --- | --- | --- | --- |
|  |  |  |  |  | **before** | **after** | **change** | **before** | **after** | **change** | **before** | **after** | **change** | **before** | **after** | **change** | **before** | **after** | **change** | **before** | **after** | **change** |
| **1** | M | 68 | 35.3 | 6 | 7.2 | 7.6 | 0.4 | 36 | 38 | 2 | 0.95 | 1.22 | 0.27 | 29 | 36 | 7 | 32 | 62 | 30 | 38 | 34 | -4 |
| **2** | M | 50 | 27.2 | 6 | 6.9 | 11.1 | 4.2 | 33 | 19 | -14 | 0.81 | 0.79 | -0.02 | 22 | 65 | 43 | 20 | 67 | 47 | 112 | 100 | -12 |
| **3** | F | 51 | 25.3 | 12 | 4.4 | 12.8 | 8.4 | 14 | 9 | -5 | 0.82 | 0.83 | 0.01 | 18 | 35 | 17 | 30 | 33 | 3 | 41 | 27 | -14 |
| **4** | M | 69 | 34.4 | 6 | 5.1 |  |  | 26 |  |  | 0.69 |  |  | 14 |  |  | 18 |  |  | 15 |  |  |
| **5** | M | 51 | 33.1 | 11 | 6.4 | 7.2 | 0.8 | 17 | 10 | -7 | 1.02 | 0.83 | -0.19 | 37 | 51 | 14 | 48 | 53 | 5 | 65 | 34 | -31 |
| **6** | M | 66 | 32.0 | 8 | 6.6 |  |  | 33 |  |  | 1.01 |  |  | 27 |  |  | 25 |  |  | 51 |  |  |
| **7** | F | 58 | 21.8 | 12 | 5.7 | 4.3 | -1.4 | 20.3 | 11 | -9.3 | 0.66 | 0.59 | -0.07 | 15 | 14 | -1 | 25 | 24 | -1 | 22 | 13 | -9 |
| **8** | F | 51 | 26.3 | 10 | 5.5 | 17.7 | 12.2 | 17.7 | 10.5 | -7.2 | 0.78 | 0.78 | 0 | 16 | 28 | 12 | 18 | 26 | 8 | 12 | 12 | 0 |
| **9** | F | 33 | 21.7 | 9 | 4.7 |  |  | 22.1 |  |  | 0.64 |  |  | 25 |  |  | 28 |  |  | 10 |  |  |
| **10** | M | 60 | 28.6 | 10 | 6.3 |  |  | 24.5 |  |  | 0.76 |  |  | 20 |  |  | 30 |  |  | 16 |  |  |
| **11** | M | 56 | 31.6 | 16 | 7 | 15 | 8 | 33 | 44 | 11 | 0.75 | 1.22 | 0.47 | 21 | 60 | 39 | 23 | 48 | 25 | 22 | 25 | 3 |
| **12** | M | 45 | 35.3 | 7 | 5.4 | 8 | 2.6 | 21 | 21 | 0 | 0.94 | 0.87 | -0.07 | 28 | 58 | 30 | 36 | 77 | 41 | 34 | 30 | -4 |
| **13** | F | 36 | ̶ | 8 | 4.8 | 7.5 | 2.7 | 13 | 9 | -4 | 0.63 | 0.66 | 0.03 | 22 | 26 | 4 | 14 | 22 | 8 | 11 | 5 | -6 |
| **14** | M | 74 | 32.0 | 14 | 8.6 |  |  | 31 |  |  | 0.88 |  |  | 25 |  |  | 19 |  |  | 34 |  |  |

**Table S6.** Electrolytes before and after long-term fasting as well as calculated changes.

| **No.** | **Sex** | **age (years)** | **BMI (kg/m^2)^** | **fasting (days)** | **sodium** | **(mmol/L)** |  | **potasium** | **(mmol/L)** |  | **magnesium** | **(mmol/L)** |  | **calcium** | **(mmol/L)** |  |
| --- | --- | --- | --- | --- | --- | --- | --- | --- | --- | --- | --- | --- | --- | --- | --- | --- |
|  |  |  |  |  | **before** | **after** | **change** | **before** | **after** | **change** | **before** | **after** | **change** | **before** | **after** | **change** |
| **1** | M | 68 | 35.3 | 6 | 140 | 140 | 0 | 3.7 | 3.9 | 0.2 | 2.05 | 2.11 | 0.06 | 9.3 | 638 | 628.7 |
| **2** | M | 50 | 27.2 | 6 | 144 | 140 | -4 | 4.1 | 4.4 | 0.3 | 2.39 | 2.33 | -0.06 | 9.1 | 9.8 | 0.7 |
| **3** | F | 51 | 25.3 | 12 | 138 | 139 | 1 | 4 | 4 | 0 | 2.32 | 2.45 | 0.13 | 9.7 | 10.3 | 0.6 |
| **4** | M | 69 | 34.4 | 6 | 139 |  |  | 4.7 |  |  | 2.15 |  |  | 9 |  |  |
| **5** | M | 51 | 33.1 | 11 | 139 | 141 | 2 | 4.2 | 4.3 | 0.1 | 1.91 | 2.04 | 0.13 | 9.7 | 9.9 | 0.2 |
| **6** | M | 66 | 32.0 | 8 | 140 |  |  | 4.5 |  |  | 2.1 |  |  | 8.9 |  |  |
| **7** | F | 58 | 21.8 | 12 | 139 | 141 | 2 | 5.9 | 4.8 | -1.1 | 0.86 | 0.89 | 0.03 | 2.53 | 2.42 | -0.11 |
| **8** | F | 51 | 26.3 | 10 | 137 | 135 | -2 | 4.8 | 4.7 | -0.1 | 0.83 | 0.79 | -0.04 | 2.41 | 2.49 | 0.08 |
| **9** | F | 33 | 21.7 | 9 | 135 |  |  | 4.5 |  |  | 0.9 |  |  | 2.25 |  |  |
| **10** | M | 60 | 28.6 | 10 | 138 |  |  | 4.3 |  |  | 0.81 |  |  | 2.27 |  |  |
| **11** | M | 56 | 31.6 | 16 | 138 | 136 | -2 | 4.2 | 4.3 | 0.1 | 1.89 | 2.56 | 0.67 | 8.7 | 9.6 | 0.9 |
| **12** | M | 45 | 35.3 | 7 | 138 | 138 | 0 | 4.4 | 4.9 | 0.5 | 1.79 | 1.95 | 0.16 | 9.2 | 9.8 | 0.6 |
| **13** | F | 36 | ̶ | 8 | 135 | 137 | 2 | 4.3 | 4 | -0.3 | 2.09 | 1.93 | -0.16 | 9 | 8.8 | -0.2 |
| **14** | M | 74 | 32.0 | 14 | 139 |  |  | 4.1 |  |  | 2.4 |  |  | 8.9 |  |  |
